# Equol-producing capacity as an overlooked modifier of pediatric isoflavone responses

**DOI:** 10.3389/fnut.2026.1993262

**Published:** 2026-10-26

**Authors:** Ruiying Fan, Yutong Xing, Kaixi Wang, Xinyu Ma, Huifeng Zhang

**Affiliations:** 1 Hebei University of Chinese Medicine, Shijiazhuang, China; 2 The Second Hospital of Hebei Medical University, Shijiazhuang, China

**Keywords:** children, gut microbiota, precision nutrition, puberty, S-equol, soy isoflavones, thyroid

## Abstract

Similar dietary isoflavone intakes need not generate similar metabolite exposures. Gut microbiota-dependent conversion of daidzein to S-equol offers a plausible, incompletely characterized modifier of pediatric endocrine responses. Pediatric observational data suggest that urinary equol may modify associations between soy intake and pubertal timing, although causality and a fixed producer phenotype have not been established. We propose treating equol-producing capacity as a developmentally dynamic metabotype and testing it through repeated functional phenotyping. Receptor-selective effects on pubertal signaling and interactions with iodine-dependent thyroid physiology remain hypotheses with unknown clinical direction. Prospective studies should integrate diet, functional metabolism, developmental stage, iodine assessment and endocrine outcomes. Current evidence does not justify equol-based dietary restriction or routine clinical testing in healthy children.

## 1 Introduction

Soy foods are widely consumed, particularly in Asian populations, yet pediatric findings on pubertal timing and thyroid indices are not uniform (1–3). Comparisons based on soy intake, isoflavone dose or formula history may overlook metabolic heterogeneity. Could gut microbiota-dependent S-equol-producing capacity represent one of the biological modifiers underlying these responses? Our central proposition is that dietary exposure should be interpreted alongside functional equol production, developmental stage, iodine status and endocrine phenotype. This Perspective therefore treats equol-producing capacity as a testable effect modifier rather than a validated clinical risk-stratification tool.

## 2 A developmentally dynamic metabotype

Intestinal microorganisms can metabolize daidzein, a soy isoflavone, to S-equol; human microbial production is stereoselective for the S-enantiomer (4, 5). S-equol has preferential binding to estrogen receptor beta (ERβ) over estrogen receptor alpha (ERα), and greater ERβ affinity than daidzein in receptor-binding assays (6). This preference is not exclusivity: transcriptional responses vary with receptor, cellular context and co-regulatory machinery (7). A single estradiol-relative potency ratio is therefore misleading. Receptor-binding affinity is not a clinical endocrine effect, and circulating metabolite concentrations do not establish receptor occupancy in a child's brain. Comparable daidzein intakes could nevertheless yield different biologically active metabolite profiles.

Adult producer prevalence estimates should not automatically be extrapolated to infants or children. In a longitudinal study of 90 infants assessed after a soy challenge, producer proportions at 6 months were 0% in the breastfed group, 3.8% in the soy-formula group and 6% in the cow-milk-formula group; production became more frequent with age (8). Among initially producing infants with subsequent observations, 66% tested negative at least once. These findings support reassessment, but the small, feeding-defined groups do not establish a causal formula or antibiotic effect. In a separate Japanese study, 13.8% of children aged 5–7 years and 35.5% of their mothers were producers after supplementation (9). A 2025 study involving 1,110 schoolchildren obtained post-supplementation urinary equol/daidzein ratios in 972 and compared production with microbiota composition in a subset (10). These observations favor age-specific assessment, not a universal pediatric prevalence. We propose a developmentally dynamic metabotype rather than a fixed lifelong binary phenotype. Microbiome maturation, diet, habitual soy intake and antibiotic exposure are candidate influences; their independent contributions to within-child changes require longitudinal testing.

Functional capacity should not be inferred from bacterial names alone. Enzymatic work in *Slackia isoflavoniconvertens* identified daidzein, dihydrodaidzein and tetrahydrodaidzein reductases (11). Comparative genomics demonstrates variation in equol-associated gene clusters, including reductase and racemase genes, across organisms (12). An *Adlercreutzia equolifaciens* strain lacking the relevant operon did not produce equol (13). Thus, neither detecting *Slackia* or *Eggerthella* nor assigning a universal “*eqg* cluster” demonstrates production. Strain-level gene content, expression, available substrate and community context separate taxonomic potential from measured metabolism.

## 3 Pubertal signaling: a directionally open hypothesis

Pubertal activation of the hypothalamic–pituitary–gonadal (HPG) axis depends on gonadotropin-releasing hormone (GnRH) network regulation, including kisspeptin and arcuate kisspeptin/neurokinin B/dynorphin (KNDy) circuitry (14). Evidence for ERα-mediated regulation is stronger than the proposed arcuate ERβ-kisspeptin pathway. In mice, deleting ERα in *Kiss1* neurons advanced pubertal onset but disrupted subsequent maturation; deleting ERα in *Tac2*-expressing neurons also advanced female pubertal development (15, 16). ERβ may contribute elsewhere: neural/glial ERβ deletion delayed female mouse puberty and reduced preoptic kisspeptin measures, without corresponding changes in the mediobasal hypothalamus (17). These experiments concern endogenous receptor function, not dietary S-equol exposure in children.

We hypothesize that an ERβ-preferential ligand might modify the balance of ERα/ERβ signaling within puberty-regulating circuits, potentially altering *Kiss1*/GnRH network activity. The direction could vary with developmental window, brain region, dose and endogenous hormonal milieu. Pediatric brain exposure at dietary concentrations remains unresolved; importantly, no direct evidence establishes dietary S-equol activation of an arcuate ERβ-*Kiss1* pathway as a cause of earlier puberty.

Human findings caution against assuming acceleration. In 4,781 Chinese children, higher soy intake was associated with later pubertal timing; associations were stronger among children with higher urinary equol in a 1,311-participant subset (2). This observation is compatible with metabolic effect modification but does not establish an equol-mediated mechanism. Importantly, urinary equol measured under habitual dietary conditions is a composite marker influenced by recent daidzein intake, timing of exposure, urinary dilution, and microbial conversion rather than a direct measure of stable equol-producing capacity. Therefore, the stronger soy–puberty association observed among children with higher urinary equol should not be interpreted as evidence that a fixed equol-producer phenotype determines pubertal timing. Future pediatric studies should distinguish measured metabolite concentration from functional producing capacity by ensuring adequate substrate exposure and repeating phenotyping over time.

Although ERα and ERβ are expressed in human growth plates (18), whether equol-producing capacity modifies skeletal maturation in children remains unknown; skeletal outcomes should therefore be considered exploratory.

## 4 Thyroid physiology: separate enzyme inhibition from estrogenicity

The thyroid hypothesis requires a distinct mechanistic argument. Genistein and daidzein inhibited thyroid peroxidase (TPO) in experimental systems; iodide protected against enzyme inactivation under specified assay conditions (19). Rats exposed to genistein showed reduced TPO activity without corresponding changes in circulating thyroid hormones or thyroid-stimulating hormone (TSH) (20). Thus, *in vitro* TPO inhibition does not automatically imply clinically meaningful human thyroid dysfunction. S-equol's receptor preference cannot establish stronger TPO inhibition than its precursor.

Iodine supply is a plausible modifier, supported by experimental isoflavone–thyroid literature, but an equol-specific pediatric interaction remains a hypothesis (21). A meta-analysis of adult randomized trials found no significant changes in free thyroid hormones and a small TSH increase of uncertain clinical importance; these findings cannot be transferred directly to children (22). Pediatric data are not absent: a study of 268 children reported modest associations between circulating parent isoflavones and thyroid measures (3). In a cross-sectional analysis including adolescents, urinary equol was inversely associated with total thyroxine in boys aged 12–19 years (23). Neither study established challenge-defined producer status, temporal causality or the proposed dietary exposure × functional capacity × iodine interaction.

The testable question is whether equol-producing capacity modifies thyroid responses to isoflavones under marginal vs. sufficient iodine conditions, not whether equol is intrinsically goitrogenic. This specific interaction has not, to our knowledge, been directly evaluated prospectively in pediatric cohorts. Children receiving levothyroxine for congenital hypothyroidism require separate consideration: soy feeding has been associated with prolonged TSH elevation during treatment, an issue involving medication management rather than demonstrated equol toxicity (24). Thyroid hormones are essential for early neurodevelopment (25); this established principle is not evidence that dietary equol causes neurodevelopmental injury.

## 5 Soy formula as an exposure model, not a presumption of harm

Soy formula can provide substantial parent-isoflavone exposure, while early infant equol production is often limited (8, 26). Formula history therefore cannot substitute for measured S-equol exposure. A longitudinal study found no developmental-score differences between soy- and cow-milk-formula-fed infants during the first year (27). Another cohort detected differences in estrogen-responsive tissue trajectories in soy-fed girls, but did not establish long-term clinical harm or an equol-specific mechanism (28). At age 14 years, a 2026 follow-up reported similar pubertal and reproductive-development measures across infant-feeding groups, although its observational design and follow-up participation limit causal inference (29). These findings do not support a simple claim of generalized endocrine damage; soy-formula cohorts remain useful developmental exposure models when metabolite production is measured rather than assumed.

## 6 A prospective pediatric research framework

A prospective pediatric framework integrating dietary exposure, functional equol phenotyping, microbiome characteristics and age-appropriate endocrine outcomes is proposed in Figure 1. A multicenter cohort recruited before puberty, with an infant subcohort where feasible, should use repeated measurements to capture both exposure and changes in metabolic capacity.

**Figure 1:**
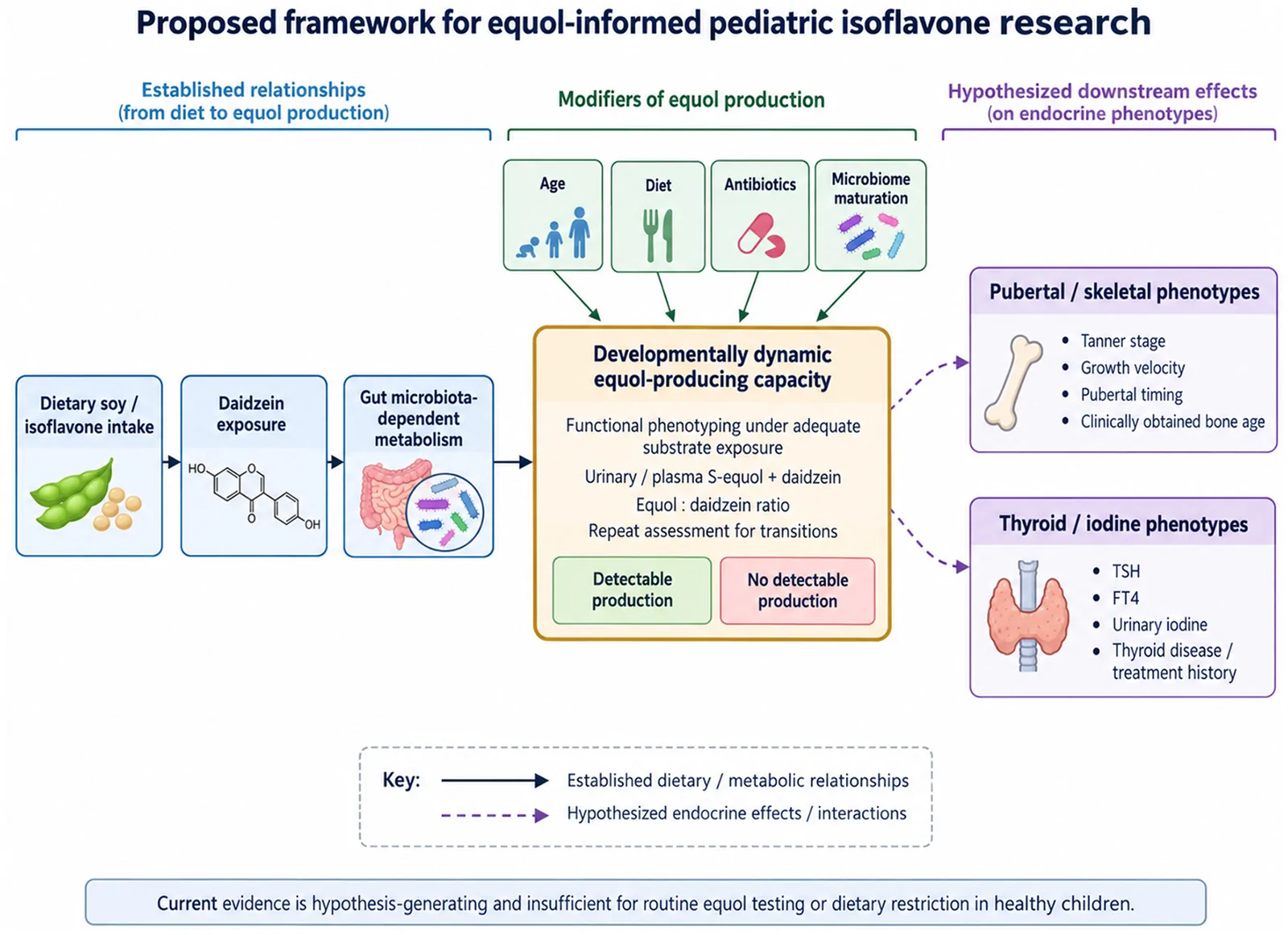
Proposed framework for equol-informed pediatric isoflavone research. Soy/isoflavone intake provides daidzein as substrate for microbiota-dependent S-equol production. Equol-producing capacity is conceptualized as a developmentally dynamic metabotype influenced by age, diet, antibiotic exposure, and microbiome maturation. Functional phenotyping distinguishes detectable from non-detectable production under adequate substrate exposure. Dark-blue solid arrows indicate established dietary or metabolic relationships; green arrows denote candidate influences on equol-producing capacity; purple dashed arrows indicate hypothesized downstream endocrine effects or interactions. The framework prioritizes longitudinal pubertal timing and thyroid/iodine phenotypes for initial testing, while skeletal outcomes are exploratory. Current evidence remains hypothesis-generating and does not support routine equol testing or metabotype-specific dietary restriction in healthy children.

Two endocrine domains should be prioritized for initial hypothesis testing: longitudinal pubertal timing and thyroid function interpreted in relation to iodine status. Pubertal outcomes should include age- and sex-appropriate milestones such as breast or testicular development, growth velocity, and menarche where applicable, whereas thyroid assessment should prioritize TSH and FT4 alongside iodine exposure. Skeletal outcomes, including clinically obtained bone age, should remain secondary or exploratory until more direct pediatric evidence is available.

The distinction between capacity and concentration is essential. Equol concentration reflects both substrate intake and conversion, whereas challenge-defined capacity is a candidate effect modifier; measured equol may also lie on the causal pathway as a mediator. These roles are not interchangeable, so interaction and mediation analyses should be prespecified separately rather than inferred from biomarker–outcome correlations.

First, characterize dietary exposure using a validated food-frequency questionnaire and, where feasible, repeated dietary records, with estimates of soy foods, soy protein and daidzein/genistein intake. Record formula history, supplements and overall dietary pattern, and distinguish whole-food matrices from isolated isoflavone supplements.

Second, prioritize functional phenotyping by measuring urinary S-equol and daidzein together, their molar ratio and, where feasible, plasma S-equol. Where ethically and developmentally appropriate, substrate exposure should be standardized using a prespecified soy or daidzein source with quantified daidzein content. Sampling should occur within the same predefined post-exposure window across participants, with consistent correction for urinary dilution and the same analytical platform whenever possible. Classification thresholds should be prespecified and age appropriate, and adequate daidzein exposure should be confirmed before assigning non-producer status. Phenotyping should be repeated at fixed longitudinal intervals and after major developmental or exposure changes, such as pubertal transition, substantial dietary change, or antibiotic exposure. Adult supplement doses or classification thresholds should not be transferred unchanged to children.

Third, pair metabolite measurements with shotgun metagenomics, targeted equol-biosynthesis genes and equol-associated taxa where resources permit. Taxonomic or gene detection is not equivalent to functional equol production and should be validated against measured conversion (12, 13).

Fourth, prioritize longitudinal pubertal outcomes using Tanner stage, growth velocity, age at breast development, testicular volume and menarche as appropriate. Gonadotropins and sex steroids require age-appropriate interpretation, and GnRH stimulation should remain clinically indicated. Skeletal measures, including bone age when clinically obtained, should be treated as exploratory rather than primary outcomes.

Fifth, prioritize thyroid function in relation to iodine status by integrating TSH, free thyroxine (FT4), urinary iodine concentration, dietary iodine, iodine-containing supplements and thyroid disease or treatment history. A single spot urinary iodine value should not classify an individual child as iodine-deficient because within-child variation can be substantial; repeated measurements or complementary dietary context improve individual interpretation (30). Thyroid antibodies should be added only when clinically or analytically justified.

Sixth, prespecify key confounders and effect modifiers, including age, sex, body mass index (BMI)/BMI standard deviation score (BMI-SDS), baseline pubertal stage, energy intake, dietary pattern, socioeconomic factors, antibiotics, probiotics, ethnicity/geography and iodine status. Repeated measures should establish temporal order and distinguish stable capacity from developmental transitions. Exposure–capacity and selected developmental or iodine interactions should be tested with adequate sample size and multiplicity control.

The primary prediction is that comparable isoflavone exposure will show different outcome associations across repeatedly characterized equol-producing capacities, conditional on developmental and nutritional context. Replicated null interactions with precise estimates would weaken this proposal, whereas consistent exposure–capacity interactions should precede any attempt to recommend equol testing or metabotype-specific dietary limits.

## 7 Discussion

Gut microbiota-dependent S-equol-producing capacity may represent an overlooked effect modifier of pediatric responses to dietary soy isoflavones. Existing observations justify testing a developmentally dynamic metabotype rather than presuming a uniformly beneficial or harmful response. Functional equol phenotyping could improve exposure characterization and support developmentally informed precision-nutrition research. Initial testing of this framework should prioritize longitudinal pubertal timing and thyroid–iodine physiology, while skeletal outcomes remain exploratory until stronger pediatric evidence becomes available. At present, available evidence is insufficient to support routine equol testing or metabotype-specific dietary restriction in healthy children.

## Data Availability

The original contributions presented in the study are included in the article/supplementary material, further inquiries can be directed to the corresponding author.
